# Nutrient Alteration Drives the Impacts of Seawater Acidification on the Bloom-Forming Dinoflagellate *Karenia mikimotoi*

**DOI:** 10.3389/fpls.2021.739159

**Published:** 2021-10-21

**Authors:** Qian Liu, Yanqun Wang, Yuanyuan Li, Yijun Li, You Wang, Bin Zhou, Zhongyuan Zhou

**Affiliations:** ^1^College of Marine Life Science, Ocean University of China, Qingdao, China; ^2^Laboratory for Marine Ecology and Environmental Science, Pilot National Laboratory for Marine Science and Technology, Qingdao, China; ^3^College of Environmental Science and Engineering, Ocean University of China, Qingdao, China; ^4^College of Life Sciences, Qingdao University, Qingdao, China

**Keywords:** seawater acidification, nutrient alteration, mitochondrial metabolism, hemolytic activity, *Karenia mikimotoi*

## Abstract

Seawater acidification and nutrient alteration are two dominant environmental factors in coastal environments that influence the dynamics and succession of marine microalgae. However, the impacts of their combination have seldom been recorded. A simulated experimental system was set up to mimic the effects of elevated acidification on a bloom-forming dinoflagellate, *Karenia mikimotoi*, exposed to different nutrient conditions, and the possible mechanism was discussed. The results showed that acidification at different pH levels of 7.6 or 7.4 significantly influenced microalgal growth (*p*<0.05) compared with the control at pH 8.0. Mitochondria, the key sites of aerobic respiration and energy production, were impaired in a pH-dependent manner, and a simultaneous alteration of reactive oxygen species (ROS) production occurred. Cytochrome c oxidase (COX) and citrate synthase (CS), two mitochondrial metabolism-related enzymes, were actively induced with acidification exposure, suggesting the involvement of the mitochondrial pathway in coping with acidification. Moreover, different nutrient statuses indicated by various N:P ratios of 7:1 (N limitation) and 52:1 (P limitation) dramatically altered the impacts of acidification compared with those exposed to an N:P ratio of 17:1 (control), microalgal growth at pH 7.4 was obviously accelerated with the elevation of the nutrient ratio compared to that at pH 8.1 (*p*<0.05), and nutrient limitations seemed beneficial for growth in acidifying conditions. The production of alkaline phosphatase (AP) and acid phosphatase (AcP), an effective index indicating the microalgal growth status, significantly increased at the same time (*p*<0.05), which further supported this speculation. However, nitrate reductase (NR) was slightly inhibited. Hemolytic toxin production showed an obvious increase as the N:P ratio increased when exposed to acidification. Taken together, mitochondrial metabolism was suspected to be involved in the process of coping with acidification, and nutrient alterations, especially P limitation, could effectively alleviate the negative impacts induced by acidification. The obtained results might be a possible explanation for the competitive fitness of *K. mikimotoi* during bloom development.

## Introduction

Phytoplankton are the dominant primary producers and bases of marine ecosystems. However, the mass occurrence of microalgal species, especially those with toxic secondary metabolites (phycotoxins), harmful algal bloom (HAB) species, impair socioeconomic interests and human health in coastal regions worldwide and thus have aroused worldwide concern (Hallegraeff, 2003). Both suddenly massive occurrence and algal toxins production are mostly the result of complex interactions between environmental changes community processes (Wohlrab et al., 2020). It has been reported that the frequency and scale of HABs have steadily increased in scenarios of global changes (Nielsen et al., 2012; Wells et al., 2015; Gobler et al., 2017), including stimulated by seawater acidification (Riebesell et al., 2018; Wells et al., 2019) and related variations in nutrients (Wells et al., 2015; Zheng et al., 2016).

Generally, seawater acidification is said to affect the physiology of some HAB species by having consequences related to growth and toxin production (Wells et al., 2015; Brandenburg et al., 2019). Whether changes in physiological performance can lead to the expansion of HAB species under seawater acidification depends on how they affect the competitive fitness of HAB species over that of other coexisting species (Riebesell et al., 2018). Seawater acidification could directly affect the carbon acquisition of HAB species, for example, the CCM of dinoflagellate was downregulated under enhanced *p*CO_2_ conditions, which could in turn result in energy reallocation from C-acquisition to other cellular processes (Bercel and Kranz, 2019; Van de Waal et al., 2019). Bercel and Kranz (2019) found that rising *p*CO_2_ levels could result in increased toxicity of *Karenia brevis* blooms. Similarly, acidification may benefit the HAB-forming ichthyotoxic raphidophyte *Heterosigma akashiwo* (Nimer et al., 1997), which was easier to obtain competitive advantage than coexisting species. Therefore, increased in bloom occurrence in a future ocean may specifically benefit from seawater acidification (Van de Waal et al., 2013; Eberlein et al., 2014).

The concentrations of nitrogen (N) and phosphorus (P), as the most essential and frequent limiting nutrients, will influence the population dynamics and physiology of harmful algae at the species level (Glibert et al., 2006). Not only the contents but also their ratios (N:P) play essential roles in this process (Guan and Li, 2017), but how these changes influence the competitive success of HABs will depend in large part on their influence on nutrient uptake. In addition, it is commonly accepted that nutrient imbalance is one of the main driving forces involved in microalgal succession through toxin production (Glibert et al., 2018), and its effect is species-specific for different HAB groups (Fu et al., 2010; Radan and Cochlan, 2018; Wang et al., 2019b). For instance, N and P limitation of growth rate substantially increases brevetoxins in *K. brevis* blooms (Hardison et al., 2014). In fact, as a driver, nutrient availability was also proven to be helpful in modulating the impacts induced by seawater acidification (Sett et al., 2014; Taucher et al., 2015). Previous studies have shown that with the simultaneous intensification of acidification and nutrients, the cell growth of *Prorocentrum donghaiense* was significantly accelerated, that is, dinoflagellate was more adaptive than diatom (Zheng et al., 2016). In addition, some investigations in temperate regions reported that the responses of plankton communities to seawater acidification are most pronounced under conditions of nutrient limitations (Bach et al., 2016; Sala et al., 2016). Very few studies, however, have focused on the combined effects of seawater acidification and nutrient limitations on the physiological responses and toxin production of HAB species (Fu et al., 2010; Sun et al., 2011), despite its obvious relevance to phytoplankton growth in the present day and future coastal ocean. This inspires a question: what are the possible mechanisms of the underlying reason for seawater acidification altering the functional responses under different nutrient conditions?

*Karenia mikimotoi* is a common and representative harmful algal species in marine ecosystems, that often forms large algal blooms in the south Atlantic and east coast of United States and European coasts, especially in Asian areas (Brand et al., 2012; Li et al., 2019). Large-scale HABs occurred almost annually in the East China Sea from 2002 until now (Sun et al., 2007; Li et al., 2017), and the dinoflagellate *K. mikimotoi* was found to be one of the causative species (Chen et al., 2021). *K. mikimotoi* is evidenced to release toxins which threaten local fisheries and the health of the food web (Brand et al., 2012; Chen et al., 2021). At present, numerous studies into the population dynamics, nutritional characteristics, and toxicological mechanisms of *K. mikimotoi* blooms have been carried out (Sun et al., 2007; Zou et al., 2010; Guan and Li, 2017; Zhao et al., 2017). However, the ecological processes related to the formation of *K. mikimotoi* blooms remain largely unclear, and it cannot fully explain the mechanism behind this phenomenon. As global climate change adds new level of instability to complex marine ecosystems, expansion and intensification trends of HAB species need to be considered regionally and at the species level (Hallegraeff et al., 2021).

Increasingly higher atmospheric CO_2_ is predicted in the near future in addition to the current imbalance of N:P ratios in coastal areas (Wang et al., 2019a; Tan et al., 2020). How does *K. mikimotoi* adapt to complex stress conditions and become dominant? Will its species dominance continue in the upcoming scenario? In addition to the series of physiological changes currently concerned that affect the competitive adaptability of HAB species, mitochondrial metabolism, as a multistep pathway that involves matrix- and membrane-associated enzymes and plays a key role in acclimation to variable environmental conditions (Leo et al., 2020). However, seawater acidification is seldom documented (Zheng et al., 2016). The above statements enabled us to perform the present study based on our previous studies that the ROS-initiated mitochondrial linked pathway was involved (Sun et al., 2016, 2017), and the effects of different pH levels when exposed to various nutrient statuses were analyzed regarding the physiological responses of *K. mikimotoi*. The present study sheds light on elucidating the adaptive strategy of *K. mikimotoi* when facing the combination of seawater acidification and nutrient limitations during the process of HAB development.

## Materials and Methods

### Microalgal Cultivation

*K. mikimotoi* (MEL22) isolated from coastal Pingtan, Fujian Province and kindly provided to us by Institute of Oceanography, Chinese Academy of Sciences. Cells were cultured in 0.45-μm filtered natural seawater, which had been autoclaved (30min, 121°C) and enriched with f/2 medium (Guillard, 1975). All cultures were incubated at 20±1°C and illuminated under a 12-h light–dark cycle with radiance of 80μmol photon m^−2^ s^−1^ provided by cool white fluorescent tubes in a constant temperature incubator. The pH and salinity were kept constant at 8.10±0.02 and 30±1.0, respectively. Flasks were manually shaken twice a day at a set time to avoid cell sedimentation. Cells in the exponential phase were used in the assays.

### Acidifying System Set-Up

The establishment of the acidifying system was based on the methods of our previous study (Hu et al., 2017; Sun et al., 2017). Three pH levels were evaluated in the present study: pH 8.1 (present ambient seawater pH, *p*CO_2_≈390ppm), pH 7.6 (predicted pH in 2100, *p*CO_2_≈1,000ppm), and pH 7.4 (predicted pH in 2300, *p*CO_2_≈2000ppm), obtained by gentle bubbling with 0.22-μm filtered ambient air and air/CO_2_ mixtures. The air/CO_2_ mixtures were generated by plant CO_2_ chambers (HP400G-D, Ruihua Instrument & Equipment Ltd., Wuhan, China) with a variation of less than 5%. The pH values and salinity of the seawater in the flasks were measured by a pH meter (Seven Compact™ S210k, Mettler Toledo, Switzerland) and a handheld salinometer (WY028Y, HUARUI, CHN).

### Experimental Design

#### Effects of Seawater Acidification

A control (pH 8.1) and two treatment groups (pH 7.6 and pH 7.4) were used in this experiment. The experiments were conducted in triplicate in 500-mL sterilized flasks containing 350mL of medium. The initial density of *K. mikimotoi* was 1×10^4^cells mL^−1^. The whole experiment lasted for 20days and was performed in triplicate for each treatment. Samples were collected 8, 12, and 15days after exposure for further physiological and biochemical analyses.

#### Effects of Seawater Acidification With Different Nutrient Conditions

Microalgae were exposed to a two-factor experimental design (2×3) with 2 pH levels of 8.1 and 7.4 and 3 nutrient levels with different N:P ratios, which were 17:1 (close to the Redfield ratio, as a control), 7:1 (N:P <16:1, as N limitation) and 52:1 (N:P >16:1, as P limitation; Table 1), and NaNO_3_ and Na_2_HPO_4_·7H_2_O were used to adjust the concentrations of N and P, respectively. All possible combinations were tested with the results of 6 treatments. The other conditions were the same as those in 2.3.1 unless otherwise stated.

**Table 1:** Seawater acidification and nutrient levels in different groups.

pH level	Nutrient level	NO_3_^−^-N (μmol/L)	PO_4_^3−^-P (μmol/L)	N:P ratios
pH 8.1	Control	35.7	1.45	17:1
	N limitation	14.3	1.45	7:1
	P limitation	35.7	0.48	52:1
pH 7.4	Control	35.7	1.45	17:1
	N limitation	14.3	1.45	7:1
	P limitation	35.7	0.48	52:1

### Assay of the Microalgal Population Dynamics

The growth of *K. mikimotoi* was determined according to the method described by Deng et al. (2017). The cell density was determined and quantitatively simulated using a logistic equation. The specific growth rate (*μ*, d^−1^) was calculated and analyzed by using the following equation (OECD, 2011):


$$\mu_{i-j}\left(d^{-1}\right)=\frac{\ln X_{j}-\ln X_{i}}{t_{j}-t_{i}}\left(d^{-1}\right)$$

where *μ_i−j_* represents the average specific growth rate from time *i* to *j*, *X_i_* represents the cell density at time *I,* and *X_j_* represents the cell density at time *j*.

We evaluated the growth performance of each strain and regressed the population growth curves using the following logistic growth model (Zhao et al., 2015):


(1) $$N_{t}=K/1+e^{a-rt}$$

(2) Tp=ar

where *N_t_* (cells ml^−1^) is the algal density at time t, *K* (cells mL^−1^) is the carrying capacity, *r* (d^−1^) is the maximum specific growth rate, and *T_p_* is the inflection point of population growth curve.

### Transmission Electron Microscopy Observation

On the 8th and 15th days after exposure, 50mL microalgal medium was sampled from the control and acidification-treated groups and centrifuged at 3000r/min. Then, the collected microalgal cells were fixed on ice with 3.5% glutaraldehyde in phosphate-buffered saline (PBS; 0.1molL^−1^, pH 7.2). The treated samples were kept at 4°C overnight in fixative liquid and washed 3 times with the same solution. Then, the cells were dehydrated sequentially in graded concentrations of ethanol based on the methods of Zhu et al. (1983), preparing the slides in triplicate. A Hitachi H-7000 (Japan) TEM was utilized to observe the ultrastructure of the algal samples.

### Determination of the ROS Levels

The ROS levels were detected by using 2'7'-dichlorofluorescein diacetate (DCFH-DA, Sigma-Aldrich) as a fluorescent probe. Briefly, 2mL culture medium in each treatment was sampled and centrifuged at 3000r/min. The supernatants were discarded, and the microalgal cells were resuspended in PBS buffer (pH 7.2). DCFH-DA was added to this suspension to a final concentration of 10μM and mixed well. The mixture was incubated at 20°C in the dark for 30min and washed twice with PBS (Zhou et al., 2019). The fluorescence value was detected by flow cytometry (Beckman Coulter Inc., CA, United States) in the FL1 channel.

### Evaluation of Key Enzyme Activities in Mitochondrial Metabolism

The cytochrome c oxidase (COX) and citrate synthase (CS) activities were measured as described in Xiao et al. (2019) with slight modifications. The collected microalgal cells were homogenized in precooled lysis buffer (4°C) by ultrasonication, the mitochondrial suspension was extracted by centrifugation at 6000r/min, and the enzyme activities of COX and CS were determined by using assay kits (Shanghai Jiemei Gene Pharmaceutical Technology Co., Ltd.; Shanghai Harling Biotechnology Co., Ltd.). The results were all expressed in mg protein/min.

### Determination of Nitrate Reductase and Phosphatase Activities

The measurement methods of nitrate reductase (NR) and phosphatase, including alkaline phosphatase (AP) and acid phosphatase (AcP), were conducted as described in Mei et al. (2013) with slight modifications. Microalgal cells were collected onto 0.45μm pore size glass fiber filter membranes (GF/F, Whatman) and homogenized in precooled lysis buffer (4°C) by the ultrasonic crushing method. The suspension was extracted by centrifugation at 6,000r/min, and the enzyme activities of NR, AP, and ACP were determined using the corresponding assay kits (Nanjing Jiancheng Bioengineering Institute, Nanjing, China). The results were all expressed in mg protein/min.

### Determination of Hemolytic Activity

The microalgal culture medium in both the control and acidification-treated groups was mixed with hemocytes of the blue mussel *Mytilis edulis* according to the method of Li et al. (2018), and the hemolytic activity was evaluated based on the hemolysis rate (Caffrey et al., 1986), which was calculated as:


$$\mathrm{Hemolysis rate}\;(\%)=\frac{\left(C_{0}{\mathrm{-C}}_{t}\right)}{C_{0}}\mathrm{\times 100}$$

where C_t_ (cell/ml) denotes the hemocyte density at time *t* (hour), C_0_ is the initial density of the hemocytes, and *t* is 24h.

### Statistical Analysis

Data analysis was performed using SPSS v. 24, and the data are expressed as the means ± standard deviation (SD). The data under every treatment conformed to a normal distribution (Shapiro–Wilk, *p*>0.05), and the variances could be considered equal (Levene’s test, *p*>0.05). The effect of pH was analyzed by one-way ANOVA (LSD test). The effects of pH, nutrient status, and their interactions were analyzed by two-way ANOVA. Moreover, significant effects of the nutrient levels at each fixed pH and significant effects of pH at each time point for each nutrient level were analyzed by one-way ANOVA (LSD test). A bivariate Pearson’s correlation analysis was performed to analyze the relationship among parameters. For all analyses, significance was assigned at the *p*<0.05 level. Figures were generated using GraphPad Prism 8.0 software.

## Results

### Influence of Seawater Acidification on *K. mikimotoi*

#### Response of Population Dynamics to Seawater Acidification

**Figure 1:**
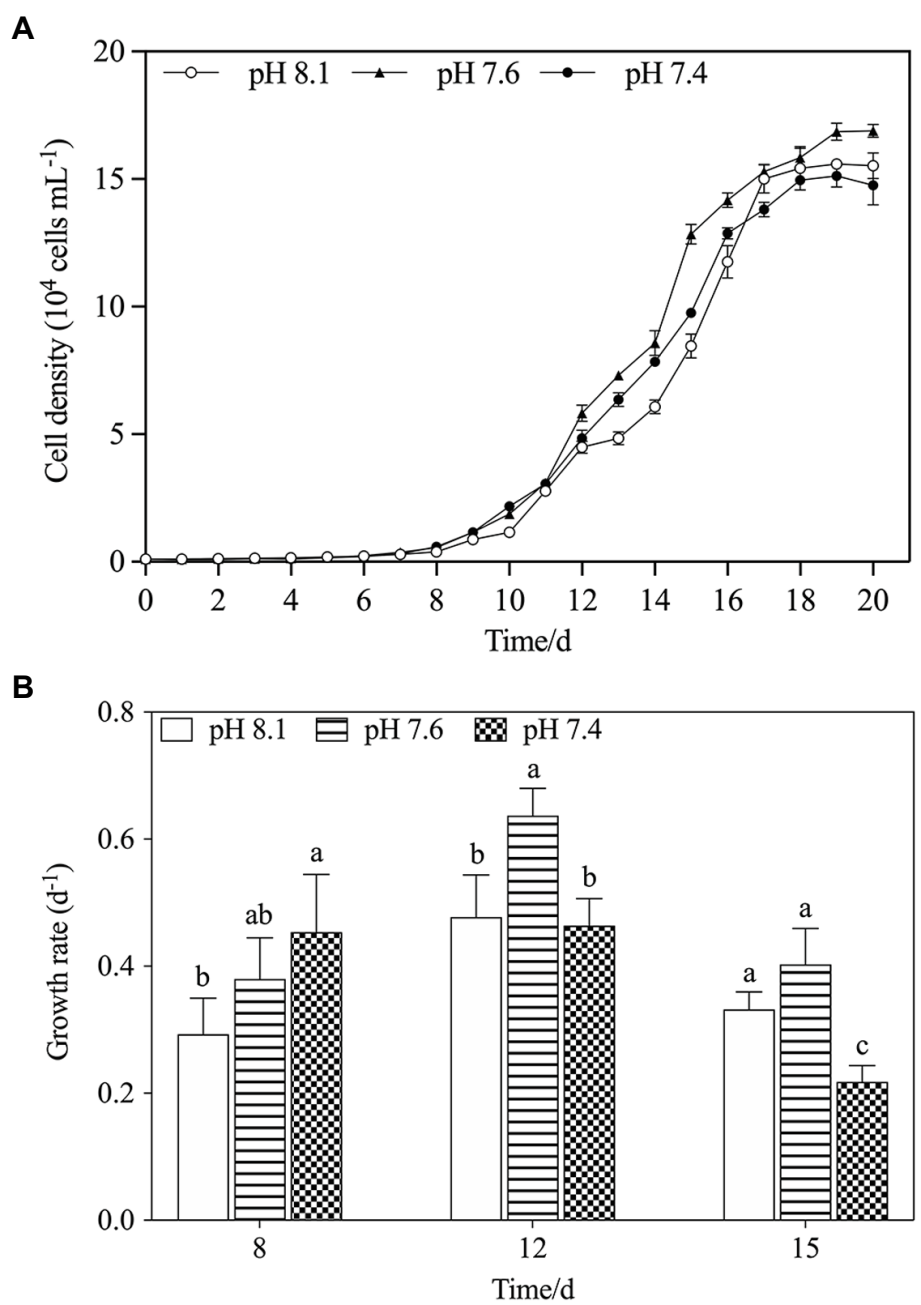
The changes of population dynamics of *K. mikimotoi* when exposed to different pH levels. **(A)** Cell density; **(B)** Specific growth rate. Data are presented as means ± SD. Different lowercase letters indicate significant differences between the treatment and the control at each time point (*p* < 0.05).

The population growth of *K. mikimotoi* under different pH levels is shown in Figure 1A. The cell density increased over time in all treatment groups and took 20days for the population to reach the stationary phase. The results of the specific growth rate (Figure 1B) showed that on the 8th day after exposure, pH 7.4 conditions promoted the growth of microalgae (*f*=6.755; *p*=0.045), but with time, compared with the pH 8.1, the pH 7.6 condition still significantly promoted the growth of the microalgae, whereas the pH 7.4 condition significantly decreased the growth rate on the 15th day after exposure (*f*=12.237; *p*=0.025). Acidification affected the growth of *K. mikimotoi* to a certain extent. We calculated the regressed parameters with a logistic model to quantify the growth dynamics in different treatments (Table 2). Based on the calculated Logistic parameters, the carrying capacity (K, reflecting the growth potential of algae) as well as the maximum instantaneous growth rate (r) in the pH 7.6 group was higher than those in the pH 8.1 and pH 7.4 group. These results suggested that *K. mikimotoi* could grow well under pH 7.6 condition.

**Table 2:** Regressions of the logistic model on *K. mikimotoi* population growth when exposed to seawater acidification conditions.

Group	*K* (×10^4^ cells mL^−1^)	*r* (d^−1^)	*Tp* (d^−1^)
pH 8.1	15.883	0.49325	14.7
pH 7.6	16.983	0.50351	13.6
pH 7.4	15.125	0.46908	13.8

#### Effects of Seawater Acidification on the *K. mikimotoi* Cellular Ultrastructure

**Figure 2:**
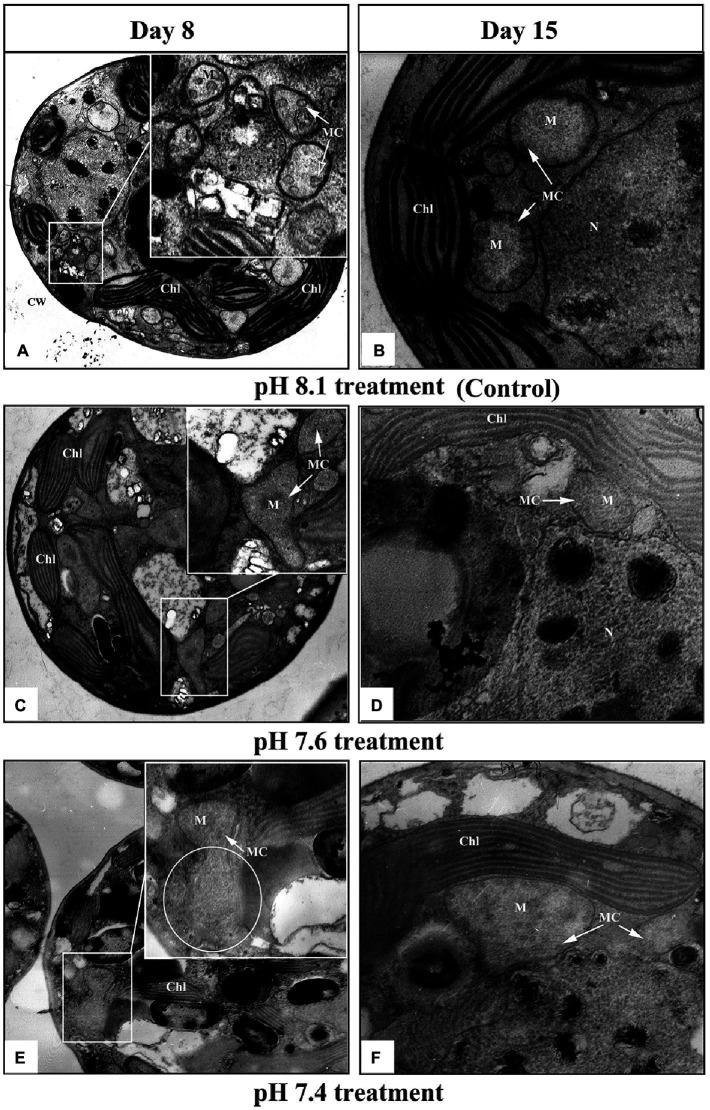
Ultrastructural changes in *K. mikimotoi* cells treated with different pH levels. **(A,B)** Control; **(C,D)** pH 7.6 treatment; **(E,F)** pH 7.4 treatment. CW, Chl, M, MC, and N in each figure denote the cell wall, chloroplast, mitochondria, mitochondrial cristae and nucleus, respectively. The scale of 2μm is indicated at the bottom of each figure.

Transmission electron microscopy images (Figure 2) showed that the mitochondria of *K. mikimotoi* cells in the pH 8.1 and pH 7.6 groups on the 8th day after exposure maintained a normal ultrastructure with intact bilayer membranes and abundant cristae (Figures 2A,C). Compared with the pH 8.1 group, obvious damage to the mitochondria of *K. mikimotoi* was observed in the pH 7.4 group, which was mainly manifested as the loss of mitochondrial inclusions, damage to the bilayer membrane, and blurring of the internal cristae (Figure 2E). On the 15th day after exposure, the mitochondrial membrane structure of the pH 8.1 and pH 7.6 groups remained intact (Figures 2B,D). Under pH 7.4 exposure, the mitochondrial membrane structures of *K. mikimotoi* were basically intact, and a few ridges had developed, showing a certain degree of recovery (Figure 2F).

#### Effects of Seawater Acidification on Mitochondrial Metabolism-Related Enzyme Activities

**Figure 3:**
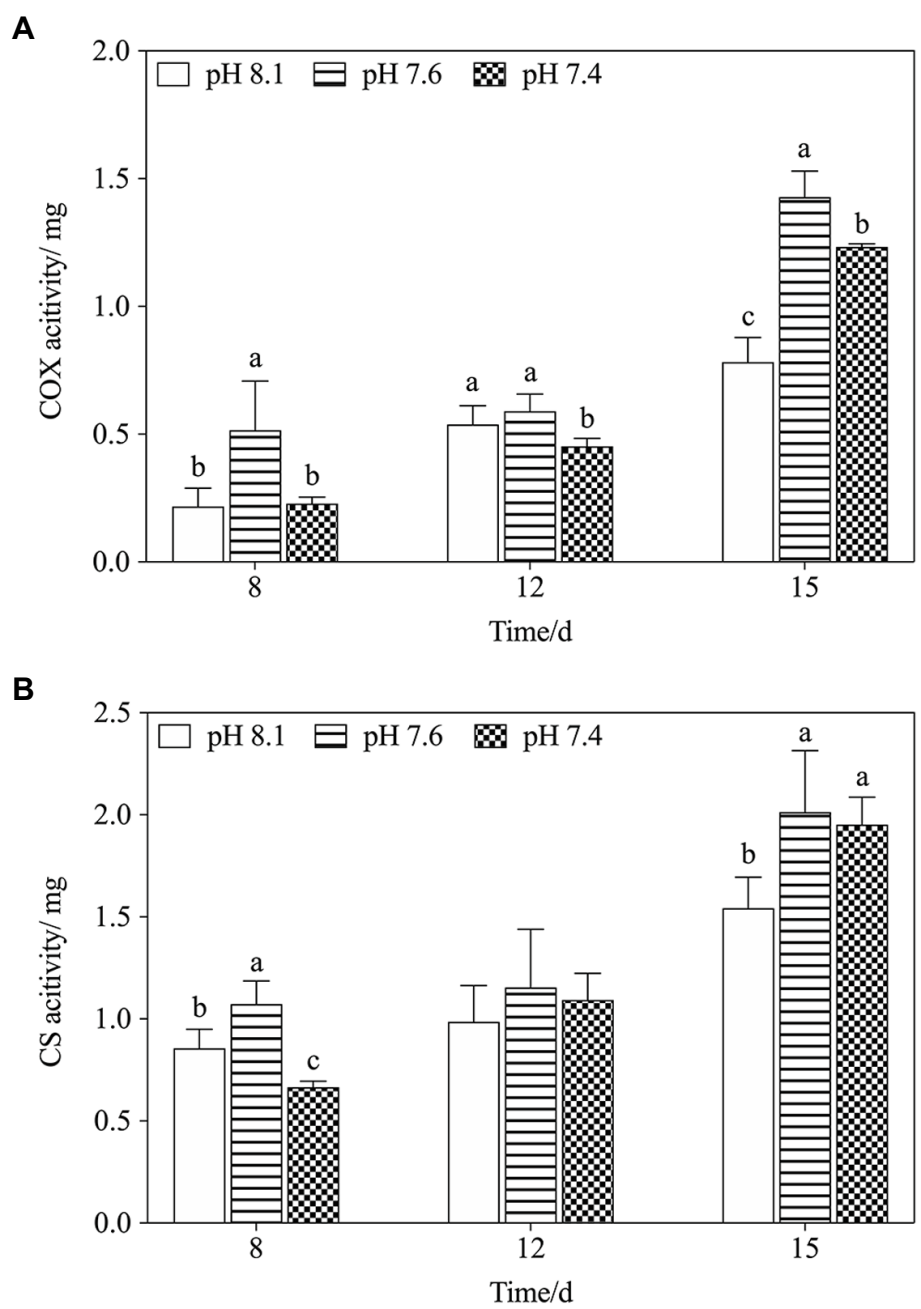
Changes in mitochondrial metabolism-related enzyme activities in *K. mikimotoi* under exposure to different pH levels. **(A)** COX activity; **(B)** CS activity. Data are presented as means±SD. Different lowercase letters indicate significant differences between the treatment and the control at each time point (*p*<0.05).

We further explored the effect of pH on mitochondrial function by measuring the activities of key mitochondrial metabolism-related enzymes (Figure 3). On the timeline, an increasing trend in COX and CS activities of all treatments was observed from day 8 to day 15 (Figures 3A,B). Exposure to pH 7.6 caused increases in the activities of COX and CS, which reached a maximum on day 15 and was significantly higher than that in the pH 8.1 group (*f*=64.125; *p*=0.0013 in COX; *f*=5.725; *p*=0.047). On the 8th and 12th days, exposure to pH 7.4 inhibited the activities of the two key enzymes to some extent; however, their activities were still higher than that in the pH 8.1group on the 15th day (*f*=27.567; *p*=0.006 in COX; *f*=12.237; *p*=0.025). During the entire exposure period, the interactive effects of pH and N/P were recorded for COX and CS activities (Table 2).

#### Effects of Seawater Acidification on ROS Production

Overall, ROS levels in *K. mikimotoi* cells increased in a pH-dependent manner, and the highest ROS content was always found in the pH 7.4 group at any set time, especially on the 8th day after exposure, which was significantly higher than that in either the pH 8.1 or the pH 7.6 group (*f*=29.133; *p*=0.000). With the extension of time, the ROS level gradually decreased and stabilized on the 12th and 15th days. The assay results indicated that ROS levels in *K. mikimotoi* could be increased by acidification.

### Influence of Seawater Acidification on *K. mikimotoi* When Exposed to Different Nutrient Statuses

#### Response of Population Dynamics to Seawater Acidification With Different Nutrient Statuses

**Figure 4:**
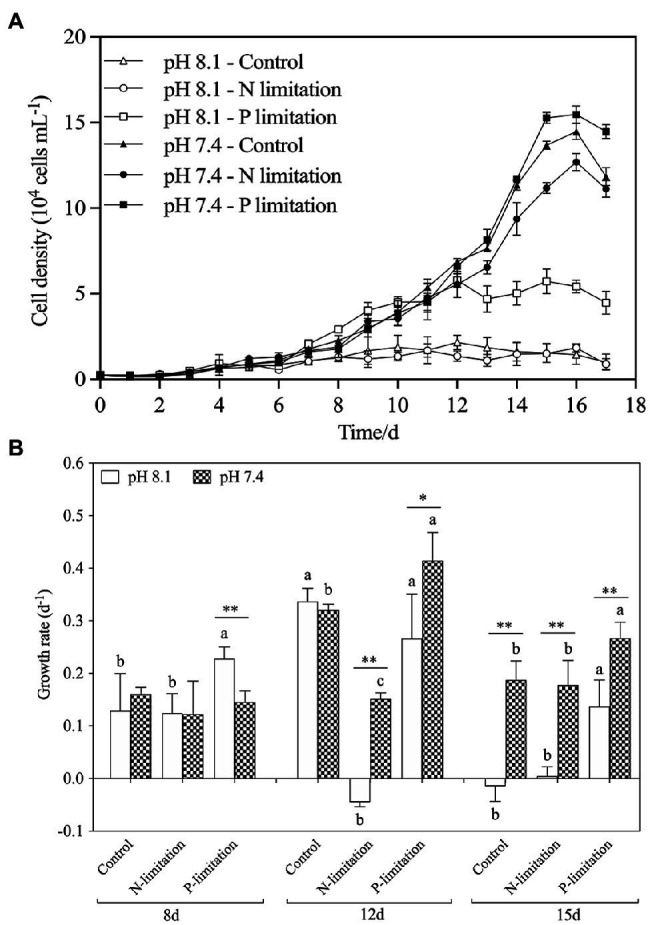
The changes of population dynamics of *K. mikimotoi* under seawater acidification conditions when exposed to different nutrient levels. Data are presented as means ± SD. **(A)** Cell density; **(B)** Specific growth rate. Different lowercase letters indicate significant differences among the three nutrient levels within each pH treatment at each time point (*p* < 0.05). Asterisks indicate significant differences between two pH levels within each nutrient treatment (*p* < 0.05).

Figure 4A showed variations of *K. mikimotoi* densities exposed to the combination of different pH and nutrient status with time. The population growth was significantly promoted by pH 7.4 with the different nutrient status, and the P limitation exhibited more increased population growth. The results of the specific growth rate (Figure 1B) showed that on the 8th day after exposure, only pH 8.1 with P limitation promoted the growth of microalgae (*f*=21.827; *p*=0.009). With the extension of time, pH 7.4 with different nutrient levels showed significant (*f*=530.72; *p*=0.000) positive effects on the population growth of *K. mikimotoi* by stimulating cell division, and the highest growth rate was found in the pH 7.4 group with P limitation on the 12th day after exposure, which was significantly higher than that in either the control or N limitation groups (*f*=6.644; *p*=0.041). Specific growth rate were significantly affected by pH, nutrient status, and their interactions during the whole experiment (Table 3). We used a logistic model to calculate the regressed parameters (Table 4). Of all the treatments, pH 7.4 with three nutrient status (control, N limitation, and P limitation) showed high *K* values, whereas microalgae grew most slowly under pH 8.1 with N limitation conditions. In addition, pH 7.4 both with control and P limitation groups showed high *r* values and long *Tp*, suggesting possible high total biomass accumulation because of the long growth period. The results indicated that the algal growth of *K. mikimotoi* was promoted significantly by acidification under different nutrient statuses.

**Table 3:** Two-way ANOVA summary on combined effects of seawater acidification (pH) and nutrient status (N/P) on ROS level, AcP activities, AP activities, growth rate, NR activities, and Hemolysis rate of *K. mikimotoi* at pH 8.1, 7.6, and 7.4; N/P: 7:1, 17:1 and 52:1.

Parameter		ROS			AcP			AP		
df		pH	N/P	pH*N/P	pH	N/P	pH*N/P	pH	N/P	pH*N/P
		1	1	2	1	1	2	1	1	2
8d	F	0.074	6.128	5.500	58.821	31.692	0.732	0.261	3.482	1.145
	P	0.790	**0.015**	**0.020**	**<0.001**	**<0.001**	0.501	0.619	0.064	0.351
12d	F	18.956	15.024	2.048	0.224	76.074	46.057	1.165	20.735	17.981
	P	**0.001**	**0.001**	0.172	0.644	**<0.001**	**<0.001**	0.302	**<0.001**	**<0.001**
15d	F	363.975	58.141	30.917	0.037	16.881	0.000	7.230	15.496	14.736
	P	**<0.001**	**<0.001**	**<0.001**	**0.005**	2.156	0.159	**0.020**	**<0.001**	**0.001**
Parameter		Growth rate			NR			Hemolysis		
df		pH	N/P	pH*N/P	pH	N/P	pH*N/P	pH	N/P	pH*N/P
		1	1	2	1	1	2	1	1	2
8d	F	88.173	40.934	35.092	11.630	0.964	0.572	88.173	40.934	35.092
	P	**<0.001**	**<0.001**	**<0.001**	**0.005**	0.409	0.579	**<0.001**	**<0.001**	**<0.001**
12d	F	5.165	23.363	44.941	0.973	10.154	1.590	0.041	4.518	14.266
	P	**0.042**	**<0.001**	**<0.001**	0.343	**0.003**	0.244	0.843	**0.034**	**0.001**
15d	F	0.365	7.968	15.887	5.976	25.777	2.371	2.703	19.721	41.455
	P	0.557	**0.006**	**<0.001**	**0.031**	**<0.001**	0.136	0.126	**<0.001**	**<0.001**

Significant effects (p < 0.05) are indicated in bold.

**Table 4:** Regressions of the logistic model on *K. mikimotoi* population growth under seawater acidification conditions when exposed to different nutrient statuses.

Group	*K* (×10^4^ cells mL^−1^)	*r* (d^−1^)	*Tp* (d^−1^)
pH 8.1			
Control	3.7721	0.4517	5.6
N limitation	2.2478	0.2438	4.7
P limitation	6.0252	0.5088	7.6
pH 7.4			
Control	15.4849	0.4868	12.3
N limitation	15.5869	0.3630	13.2
P limitation	18.2281	0.5794	12.9

#### Effects of Seawater Acidification With Different Nutrient Statuses on Nutrient Absorption Capacity

**Figure 5:**
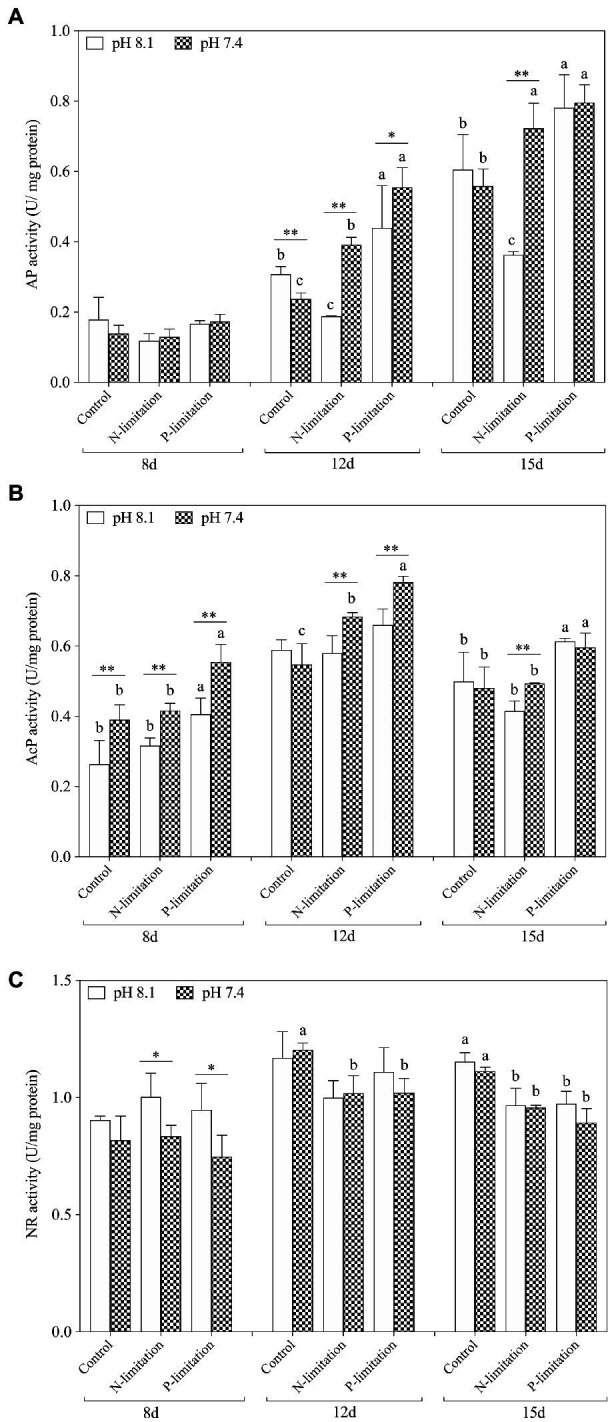
Changes in nutrient absorption-related enzyme activities in *K. mikimotoi* under seawater acidification conditions when exposed to different nutrient levels. **(A)** AP activity; **(B)** AcP activity; **(C)** NR activity. Data are presented as means±SD. Different lowercase letters indicate significant differences between three nutrient levels within each pH treatment at each time point (*p*<0.05). Asterisks indicate significant differences between two pH levels within each nutrient treatment (*p*<0.05).

The activities of AP in each treatment group increased with time (Figure 5A) and reached a peak on the 15th day after exposure, with the highest point at pH 7.4 with P limitation. The activity of ACP (Figure 5B) showed a similar trend, but the peak value occurred on the 12th day. The activities of AP and AcP in *K. mikimotoi* increased with decreasing phosphate concentration (Figures 5A,B), and significance (*f*=18.753; *p*=0.012 in ACP; *f*=2.221; *p*=0.021, in AP) was found at pH 7.4 compared to the control of pH 8.1. Specifically, the AP and AcP activities in the P limitation group were much higher than those in the control at the same pH of 7.4, followed by the N limitation group. The results indicated that both low phosphorus and low nitrogen status induced the massive increase of AP and AcP activities under acidification conditions. AP activities were significantly affected by nutrient status and their interactions at day 12 and day15, and ACP activities were only significantly affected by their interactions at day 12.

On the 8th day after exposure, pH 8.1 with N and P limitations showed a significant positive effect on NR activities compared to pH 7.4. With the extension of time (Figure 5C), the NR activities under N and P limitations were lower than those in the control on the 12th and 15th days, and there was no significant change between different pH levels under the same nutrient status. These results indicated that the effect of nutrient limitations on the activity of NR was more dramatic than that of acidification. During the whole experiment, NR activities showed no interactive significant effect. At day 8 and day 15, NR activities were significantly affected by pH and were only affected by nutrient status at day 12 and day15.

#### Effects of Seawater Acidification With Different Nutrient Statuses on ROS Production

**Figure 6:**
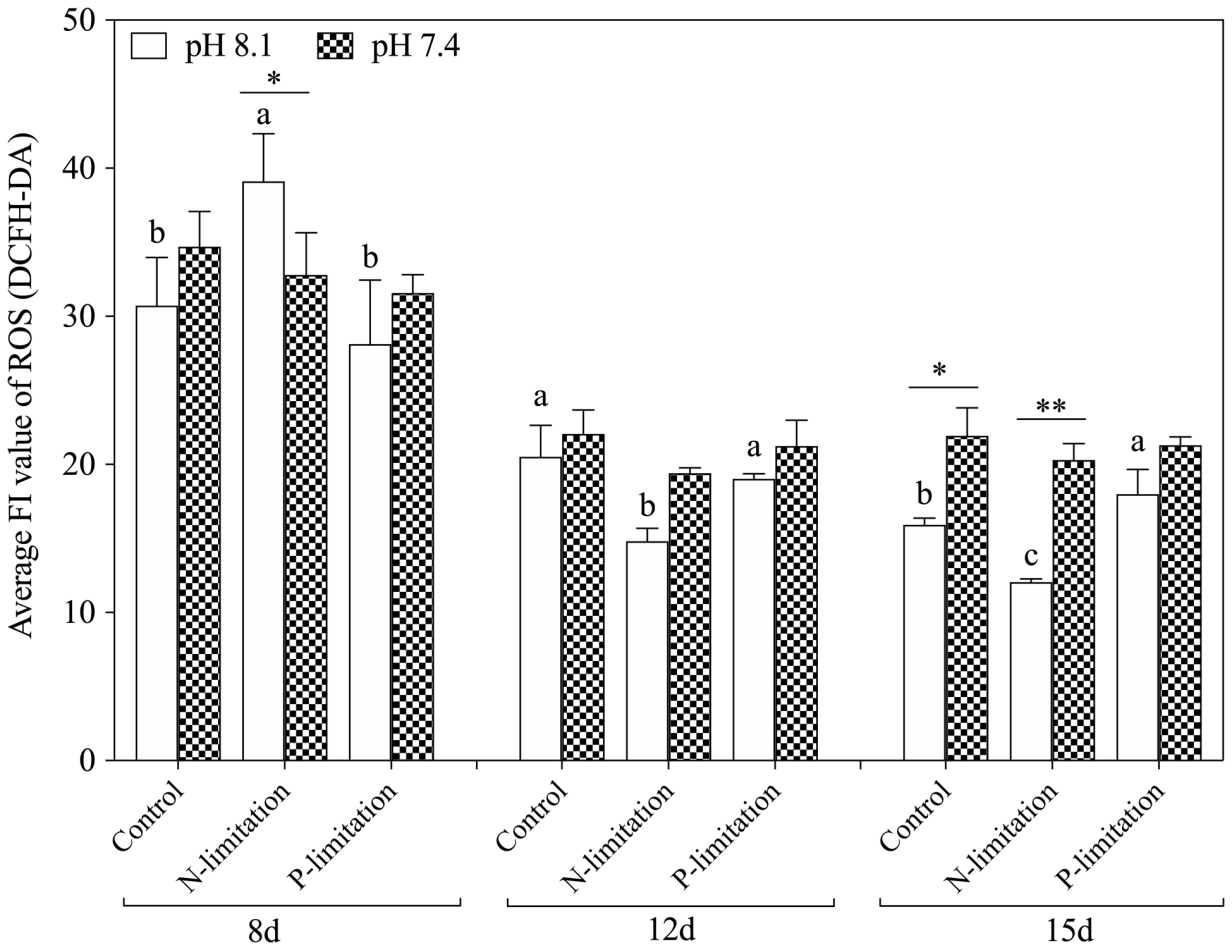
Changes in ROS levels in *K. mikimotoi* under seawater acidification conditions when exposed to different nutrient levels. Data are presented as means±SD. Different lowercase letters indicate significant differences among three nutrient levels within each pH treatment at each time point (*p*<0.05). Asterisks indicate significant differences between two pH levels within each nutrient treatment (*p*<0.05).

The relative DCF fluorescence demonstrated that the intracellular ROS level was elevated on the 8th day of exposure in each treatment group (Figure 6), and the ROS level in the pH 8.1 with N limitation group reached the highest point (*f*=70.54; *p*=0.001). With time, the ROS level decreased and then stabilized on the 12th and 15th days. In addition, the ROS levels of all nutrient treatment groups at pH 7.4 were higher than those at pH 8.1 on the 15th day (*f*=31.62; *p*=0.001). It is worth noting that the N and P limitation groups at pH 7.4 had no significant difference (*f*=1.205; *p*=0.363) in the ROS levels of *K. mikimotoi* compared with the control. The ROS levels showed higher sensitivity to acidification conditions when exposed to different nutrient statuses. ROS levels were significantly affected by nutrient status during the whole experiment. Moreover, interactive effects of pH and nutrient status were observed at day 8 and day 15 (Table 3).

#### Toxicity Responses of *K. mikimotoi* to Seawater Acidification With Different Nutrient Statuses

**Figure 7:**
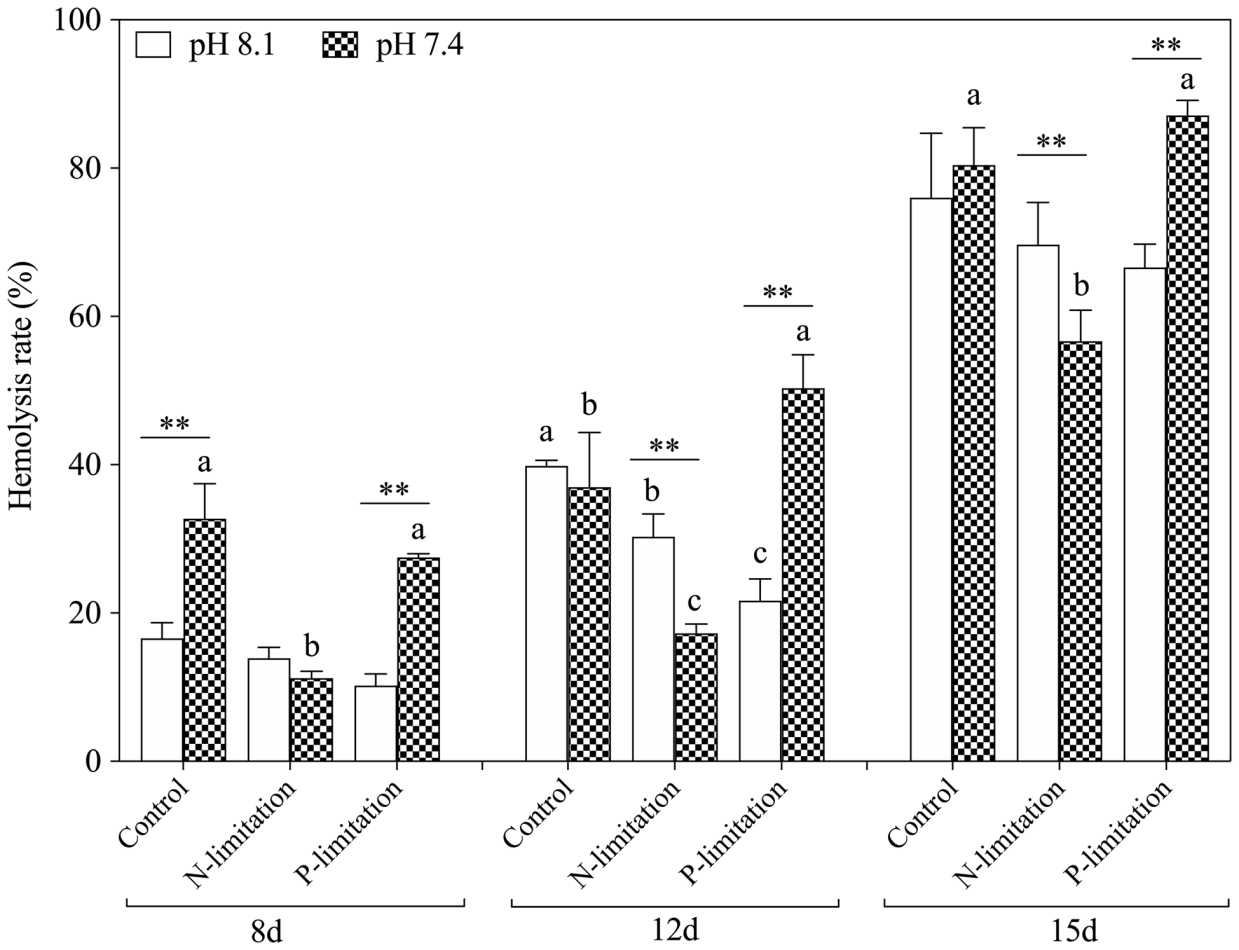
Changes in hemolytic activity in *K. mikimotoi* under seawater acidification conditions when exposed to different nutrient levels. Data are presented as means±SD. Different lowercase letters indicate significant differences among three nutrient levels within each pH treatment at each time point (*p*<0.05). Asterisks indicate significant differences between two pH levels within each nutrient treatment (*p*<0.05).

In general, the hemolysis rate increased with time (Figure 7) and reached a peak on the 15th day after exposure, with the highest point at pH 7.4 with P limitation. Specifically, the hemolysis rate in the P limitation group was much higher than that in the N limitation group at the same pH level, suggesting that *K. mikimotoi* has more adaptability to low phosphate conditions. Interestingly, N limitation conditions inhibited growth but obviously induced hemolytic activity in *K. mikimotoi* at pH 8.1. The results showed that compared to N limitation, P limitation significantly (*f*=70.541; p=0.001) enhanced the hemolysis rate in hemocytes of mussels under acidification conditions. Hemolysis rates were significantly affected by nutrient status and their interactions during the whole experiment and were only significantly affected by pH at day 8 (Table 3).

## Discussion

The present study elucidated the impact of acidification on the population dynamics of *K. mikimotoi*, and a possible explanation was proposed from a mitochondrial-metabolic perspective. In addition, the effects of acidification interacting with different nutrient statuses were discussed.

### Mitochondrial Metabolism Was Speculated to Alter During the Process of *K. mikimotoi* Coping With Seawater Acidification

In the present study, acidification (pH 7.6) promoted a certain increase in algal growth, but there was no significant stimulating effect at pH 7.4, and even inhibited the growth of algal to some extent (*p*<0.05). There are two possible explanations for this: First, the cells reallocate intercellular energy for other metabolic activities (Shi et al., 2015) and reduce it for cellular division, and second, acidification impairs the cellular structure (Shi et al., 2009), and this results in a disorder of cell physiological functions (Rost et al., 2010). Mitochondria are organelles responsible for energy production that is essential for cell division and growth (Nunes-Nesi et al., 2014). Energy synthesis in plant mitochondria originates from a sequential set of metabolic processes and then undergoes a series of enzymatic and nonenzymatic reactions to synthesize ATP *via* the tricarboxylic acid cycle (TAC) and oxidative phosphorylation (Jacoby et al., 2012). We measured the activities of two respiratory enzymes, COX and CS, which play key roles in TAC and oxidative phosphorylation and found that pH 7.6 significantly increased their activities (*p*<0.05). The activation of these enzymes facilitates ATP synthesis and increases ATP production (Xiao et al., 2019). However, pH 7.4 inhibited the activities of COX and CS on the 8th day, indicating an insufficient supply of ATP, which might disrupt cell function (Chivasa et al., 2005). The normal process of cellular biochemical reactions is based on an intact cellular structure, and interference with enzymatic activities could be due to mitochondrial structural damage (Xiao et al., 2019). TEM observations (Figure 2) showed that the mitochondria in *K. mikimotoi* cells exhibited normal morphological structures when exposed to pH 7.6 conditions (Figure 2C); therefore, the activities of these enzymes were not negatively affected. However, pH 7.4 destroyed the mitochondrial ultrastructure, damaging the bilayer membrane structure and blurring the cristae (Figure 2E). Therefore, changes in the mitochondrial structure at pH 7.4 may affect key mitochondrial enzyme activities and ATP synthesis.

**Figure 8:**
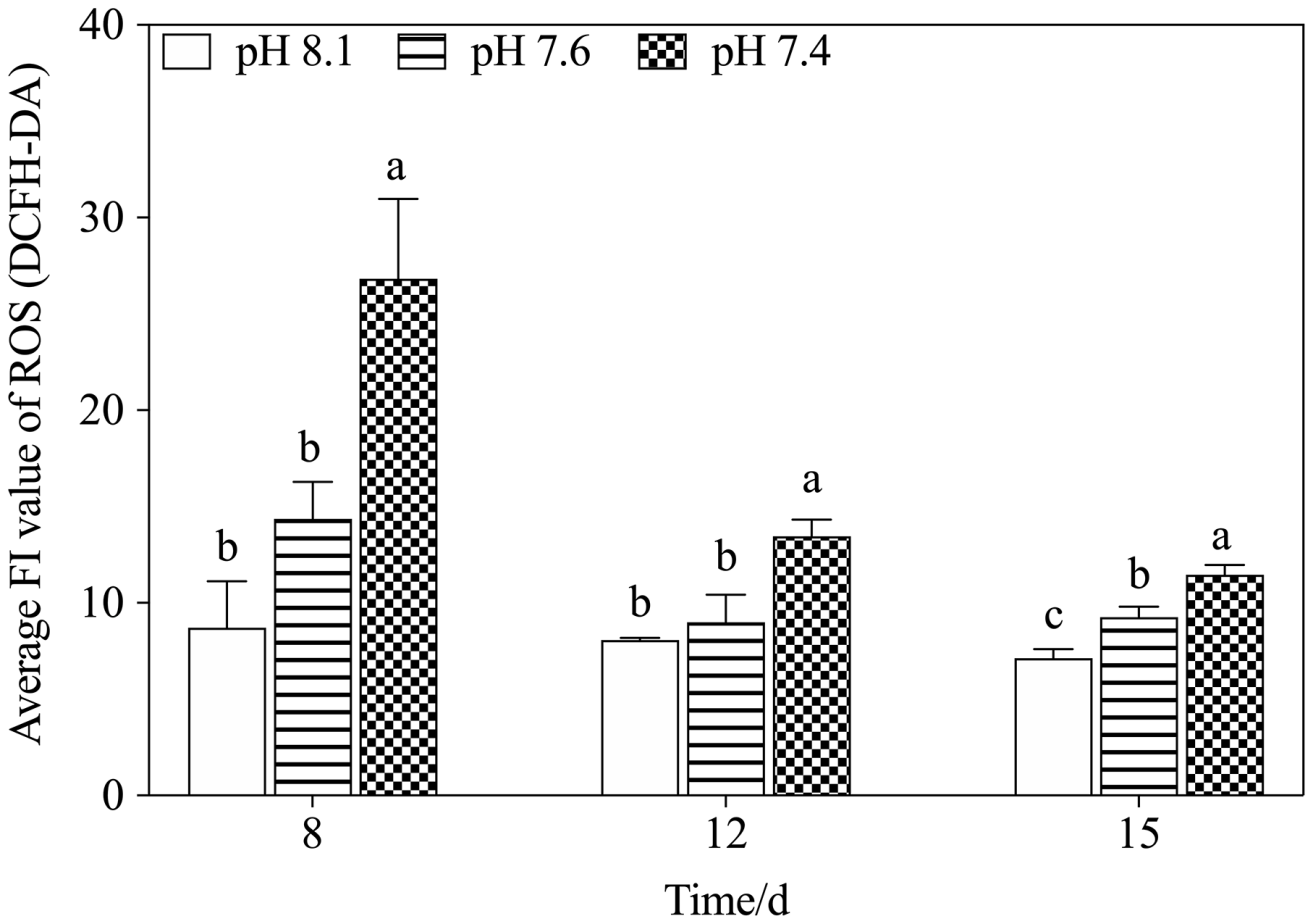
Changes in ROS levels in *K. mikimotoi* under exposure to different pH levels. Data are presented as means±SD. Different lowercase letters indicate significant differences between the treatment and the control at each time point (*p*<0.05).

Mitochondria are also responsible for ROS overproduction when aerobic respiration is blocked under stress (Ali et al., 2016; Zhang et al., 2018). It has been reported that acidification stress leads to the accumulation of ROS and results in oxidative damage (Li and Xing, 2011), and we observed similar results in the present study: pH 7.6 induced a slight increase in ROS levels and pH 7.4 stress caused ROS bursts (Figure 8), which is an important response of *K. mikimotoi* to acidification exposure on the 8th day, suggesting that pH 7.4 induces mitochondrial oxidative stress. The elevated ROS level damaged the mitochondrial ultrastructure and caused mitochondrial dysfunction in *K. mikimotoi*, which further aggravated the damage. ROS have also been shown to have direct inhibitory effects on a variety of mitochondrial enzymes, including components of the electron transport chain (Chen et al., 2005; Navrot et al., 2007). In this study, ROS were found to have a significantly negative correlation with both COX and CS in all treated groups, and this reached extreme significance (*p*<0.01) in the pH 7.4 group (Table 5). Specifically, Guedouari et al. (2014) found that temporary oxidative stress is associated with an increase in ROS production, which may constitute a signal for adaptive strategies.

**Table 5:** Pearson’s correlation coefficients between key enzyme activities and ROS levels in *K. mikimotoi* treated with seawater acidification.

		ROS	COX
pH 8.1	COX	−0.983^**^	
	CS	−0.971^**^	0.912^**^
pH 7.6	COX	−0.524^*^	
	CS	−0.529^*^	0.992^**^
pH 7.4	COX	−0.756^**^	
	CS	−0.828^**^	0.993^**^

* Significant differences at the *p*<0.05 level.

** Significant differences at the *p*<0.01 level.

It was speculated that the enzymes COX and CS, which are involved in mitochondrial metabolism, are closely related to physiological adaptability at the individual level (Ren et al., 2010). CS activation drives the TCA cycle toward a more energetic metabolism and is necessary to respond to the energetic needs of the cell. Moreover, a significant increase in COX activity may compensate for the inhibition of mitochondria (Guedouari et al., 2014). In this study, the concomitant activation of COX and CS in the pH 7.4 group reflected a shift of the algal cells toward higher levels of energy production on the 15th day, possibly providing the mitochondrial respiratory chain with a larger number of electrons. In addition, activating the key enzymes in mitochondrial metabolism helped to increase ATP production in the algal cells under acidification conditions to run some energy consumption adaptation mechanisms, including activating antioxidant systems to eliminate excessive ROS (Ali et al., 2016; Zhang et al., 2018), thus alleviating ROS damage to mitochondria. As stated above, we thus speculated that mitochondrial metabolism takes part in the active response of *K. mikimotoi* to acidification.

### Nutrient Alterations Could Drive the Effects of Seawater Acidification on *K. mikimotoi*

We found that a moderate decrease in pH could promote an increase in algal cell density, but a further decrease in pH would damage the cellular structure and function. N and P are the most crucial factors in the growth rate, and algal density increases with increasing N:P ratios in a certain range (Li et al., 2014). It is suggested that high nitrogenous nutrient availability is a prerequisite for *K. mikimotoi* blooms (Chang and Carpenter, 1985). In our study, the condition of acidification with different nutrient statuses significantly increased the population density of *K. mikimotoi*. The growth rate of *K. mikimotoi* was highly sensitive to acidification driven by nutrient alteration, especially under P limitation on the 12th and 15th days (Figure 4). Similar results found that the cell density and growth rate of *Conticribra weissflogii* and *Prorocentrum donghaiense* were increased when N/P increase under acidification conditions (Zheng et al., 2016), which was evidenced that the growth rate was controlled not only by the pH but also by the ratio of N to P. Two enzymes closely related to phosphate absorption, AP and AcP, were found to be actively induced simultaneously, and a relatively clear negative correlation was found between the enzymatic activity and the P concentration (Figure 5). This result might be a possible explanation for the population dynamics observation of the abovementioned factors and was also consistent with the following statement: acidification promotes the expression of phytoplankton phosphorus deficiency signals, thereby facilitating the absorption and utilization of phosphorus (Ivančić et al., 2010; Fitzsimons et al., 2020). However, the changes in the activities of the two phosphatases differed when exposed to N limitation conditions with acidification. The results showed that N limitation induced a progressive increase in AP activity in a time-dependent manner but only a slight increase in AcP activity on the 12th day (*p*<0.05). Therefore, both N and P limitations induced an increase in phosphatase activities in *K. mikimotoi* cells under acidification.

Increasing the activity of AP and AcP is one of the important adaptive strategies of plants to enhance P acquisition and utilization (Wang et al., 2009). It is reported that *K. mikimotoi* grows better as nitrate supplied as the only nitrogen source (Lei and Lü, 2011). However, the NR characteristics of different algae are different, which leads to differences in the nitrate utilization efficiency of algae (Berges and Hageman, 1997). In addition, the exact mechanism of nitrate on *K. mikimotoi* algal cell metabolism is still unknown. Our results showed that NR activities in *K. mikimotoi* induced by N and P limitations decreased slightly when exposed to acidification, but the difference between the two nutrient limitations was not significant. We suspect that in the process of external nitrate depletion or after exhaustion, *K. mikimotoi* utilized intracellular reserves of nitrate to maintain a certain NR activity under extremely low nitrate levels in the external environment (Dortch et al., 1979).

Hemolytic toxicity is another effective index indicating the growth status of *K. mikimotoi* (Li et al., 2019), which varies according to the growth stage and nutrient condition of *K. mikimotoi* (Lin et al., 2015). Under nutrient-sufficiency conditions, toxin production is often low, while increased production is associated with different types of nutrient limitation stress. The hemocytes of the blue mussel *M. edulis* were utilized in the present study to determine the hemolytic toxicity, which was different from the routine method that utilizes bovine or rabbit blood cells. We found that the hemolytic activity was increased in a time- and N:P ratio-dependent manner (Figure 7), and a significant elevation was seen under P limitation. These results suggest P limitation is an important factor regulating cellular toxicity and adverse impacts. Acidification (pH) and nutrient alteration (N/P) showed statistically (MANOVA) significant interactive effects on hemolytic activity (Table 3). The algae cells under high CO_2_ and P limitation conditions were the most toxic (Figure 7). Similar results showed that cytotoxicity of *Alexandrium catenella* was observed to be significantly increased under P limitation, while acidification conditions further exacerbated this toxicity (Tatters et al., 2013). Significantly, the increase in hemolytic activity is supported by N released within the cell from protein turnover, which was affected by P limitation, so that hemolytic cytotoxin synthesis was enhanced. Negative significance was also observed between hemolytic toxicity and ROS (*p*=−0.586*; Table 6). Considering the downregulation of ROS levels over time, we speculated that more energy produced by mitochondrial metabolism was allocated to the accumulation of toxic compounds (Jin et al., 2015), which enhanced the hemolytic activity during exposure to acidification with different nutrient statuses (Wang et al., 2019a). These data further explain why *K. mikimotoi* becomes the dominant species under red tide conditions by enhancing its absorption rate of nutrients and its toxicity.

**Table 6:** Pearson’s correlation coefficients among parameters under seawater acidification conditions when exposed to different nutrient statuses.

	ROS	AP	AcP	NR
pH 8.1				
ROS				
AP	−0.539^*^			
AcP	−0.641^**^	0.521^*^		
NR	−0.278	0.282	0.530^*^	
Hemolysis rate	−0.708^**^	0.727^**^	0.3	0.216
pH 7.4				
ROS				
AP	−0.745^**^			
AcP	−0.610^*^	0.589^*^		
NR	−0.562^*^	0.172	0.182	
Hemolysis rate	−0.586^*^	0.799^**^	0.009	0.202

* Significant differences at the *p*<0.05 level.

** Significant differences at the *p*<0.01 level.

## Conclusion

Taken together, seawater acidification plays a critical role in influencing the growth of *K. mikimotoi*, and mitochondrial metabolism is involved in the process of coping with acidification. Nutrient limitations, especially P limitation, could effectively alleviate the negative impacts induced by acidification, which is one of the competitive strategies used by *K. mikimotoi*. Exposure to acidification with different nutrient statuses would lead to changes in the secretion of toxins in *K. mikimotoi*, which is closely related to the formation of red tides under natural conditions.

## Data Availability Statement

The raw data supporting the conclusions of this article will be made available by the authors, without undue reservation.

## Author Contributions

YoW conceived and supervised the project. QL, YaW, and BZ conceived and designed the experiments. QL, YaW, YuL, and YiL performed the experiments. QL and YaW analyzed the data, wrote the manuscript, and contributed to this work. ZZ revised the final manuscript, figures, and tables with input from QL. All authors participated in the discussions of the results and the preparation of the manuscript. All authors contributed to the article and approved the submitted version.

## Funding

This work was financially supported by the National Key R&D Program of China (No. 2017YFC1404304) and the Fundamental Research Funds for the Central Universities (Nos. 201964024 and 202066001).

## Conflict of Interest

The authors declare that the research was conducted in the absence of any commercial or financial relationships that could be construed as a potential conflict of interest.

## Publisher’s Note

All claims expressed in this article are solely those of the authors and do not necessarily represent those of their affiliated organizations, or those of the publisher, the editors and the reviewers. Any product that may be evaluated in this article, or claim that may be made by its manufacturer, is not guaranteed or endorsed by the publisher.
